# Wedelolactone enhances osteoblastogenesis by regulating Wnt/β-catenin signaling pathway but suppresses osteoclastogenesis by NF-κB/c-fos/NFATc1 pathway

**DOI:** 10.1038/srep32260

**Published:** 2016-08-25

**Authors:** Yan-Qiu Liu, Zhi-Lai Hong, Li-Bin Zhan, Hui-Ying Chu, Xiao-Zhe Zhang, Guo-Hui Li

**Affiliations:** 1 Academy of Integrative Medicine, Dalian Medical University, Dalian 116044, China; 2 Dalian Institute of Chemical Physics, Chinese Academy of Sciences. Dalian 116023, China; 3 School of Basic Medical Sciences, Nanjing University of Chinese Medicine, Nanjing 210023, China

## Abstract

Bone homeostasis is maintained by formation and destruction of bone, which are two processes tightly coupled and controlled. Targeting both stimulation on bone formation and suppression on bone resorption becomes a promising strategy for treating osteoporosis. In this study, we examined the effect of wedelolactone, a natural product from *Ecliptae herba*, on osteoblastogenesis as well as osteoclastogenesis. In mouse bone marrow mesenchymal stem cells (BMSC), wedelolactone stimulated osteoblast differentiation and bone mineralization. At the molecular level, wedelolactone directly inhibited GSK3β activity and enhanced the phosphorylation of GSK3β, thereafter stimulated the nuclear translocation of β-catenin and runx2. The expression of osteoblastogenesis-related marker gene including osteorix, osteocalcin and runx2 increased. At the same concentration range, wedelolactone inhibited RANKL-induced preosteoclastic RAW264.7 actin-ring formation and bone resorption pits. Further, wedelolactone blocked NF-kB/p65 phosphorylation and abrogated the NFATc1 nuclear translocation. As a result, osteoclastogenesis-related marker gene expression decreased, including c-src, c-fos, and cathepsin K. In ovariectomized mice, administration of wedelolactone prevented ovariectomy-induced bone loss by enhancing osteoblast activity and inhibiting osteoclast activity. Together, these data demonstrated that wedelolactone facilitated osteoblastogenesis through Wnt/GSK3β/β-catenin signaling pathway and suppressed RANKL-induced osteoclastogenesis through NF-κB/c-fos/NFATc1 pathway. These results suggested that wedelolacone could be a novel dual functional therapeutic agent for osteoporosis.

Bone homeostasis is balanced by osteoblast-mediated bone formation and osteoclast-mediated bone resorption. Deficiency in bone formation by osteoblasts or excessive bone resorption by osteoclasts can cause metabolic bone disorders such as osteoporosis. Drugs that either increase bone building or block bone degradation are developed for treatment of osteoporosis. However, treatment with antiresorptive agents such as bisphosphonates for a long term, might lead to a simultaneous decrease in bone formation^1^,^2^,^3^. For the anabolic agent parathyroid hormone (PTH), a concomitant increase in bone resorption can be observed^4^,^5^. These drawbacks of the current therapies might be attributed to one target for these drugs that fail to uncouple bone degradation and formation: they stimulate or inhibit both processes at the same time. Research is currently focusing on drugs that can simultaneously regulate bone resorption and bone formation, and could therefore develop a new class of dual-action therapeutic agents for osteoporosis^6^,^7^.

*Ecliptae herba*, also known as “Mo-Han-Lian”, has been used as “Kidney-nourishing” traditional Chinese medicine for several thousand years. In China, “Kidney-nourishing” herbal drugs are commonly believed to have the ability of nourishing bones, and therefore are used to treat bone diseases such as osteoporosis. Recently, it was reported that *Ecliptae herba* extract showed therapeutic effect on bone metabolism of ovariectomized rats^8^,^9^. Wedelolactone is a compound isolated from *Ecliptae herba,* which has attracted much interest owing to its many biological activities, such as anti-cancer, anti-inflammatory, and antioxidant activities^10^,^11^,^12^,^13^. Our previous study showed that wedelolactone inhibited preosteoclastic proliferation and differentiation^14^. In this study we aimed to examine the effects of wedelolactone on osteclast-mediated bone resorption and BMSC-mediated osteoblast differentiation, and then elucidated the molecular mechanisms of its dual action.

Osteoblasts are derived from BMSC. Significant progress has been made over the past decade in our understanding of the molecular framework that controls BMSC differentiation towards osteoblasts. A large number of factors have been implicated in regulating osteoblast differentiation, including the Wnt family^15^. Canonical Wnt signaling is crucial for regulation of osteoblast development including osteoblast proliferation, differentiation, and survival. Activation of the canonical Wnt signaling pathway involves recruitment of a complex including LRP5/6 and GSK-3β, stabilization of β-catenin, regulation of transcription factors such as runx2, and activation of Wnt target genes^16^,^17^. This pathway is active in BMSC and therefore many signaling molecules are developed as drug targets such as GSK-3β and LRP5/6^6^.

Osteoclasts are monocyte-macrophage lineage-derived large multinucleated cells that play essential physiologic functions during bone development. The osteoclast precursors can differentiate into osteoclasts under the stimulation of cytokines such as receptor activator NF-κB ligand (RANKL) and macrophage colony-stimulating factor (M-CSF). After binding with the receptor of RANK, RANKL activates many signaling pathways, including nuclear factor-κB (NF-κB), and nuclear factor of activated T-cells cytoplasmic 1 (NFATc1)^18^,^19^,^20^,^21^. NFATc1 then translocates to the nucleus and activates the expression of multiple osteoclastogenesis-related genes, such as cathepsin K, c-Src, and tartrate-resistant acid phosphatase (TRAP).

In the present study, we investigate the effect of wedelolactone on osteoblastogenesis and osteoclastogenesis *in vitro* and *in vivo*, and elucidate its molecular mechanisms underlying. We observed that wedelolactone stimulated BMSC differentiation into osteoblasts through activation Wnt/GSK3β/β-catenin pathway. At the same concentration range, wedelolactone inhibited osteoclastic RAW264.7 activity by inhibiting RANK/NF-κB/c-fos pathway. The bone formation stimulatory and bone resorption inhibitory activity of wedelolactone was further verified in ovarietomy-induced bone loss *in vivo*.

## Results

### Wedeloloactone enhances BMSC differentiation towards osteoblasts

**Figure 1:**
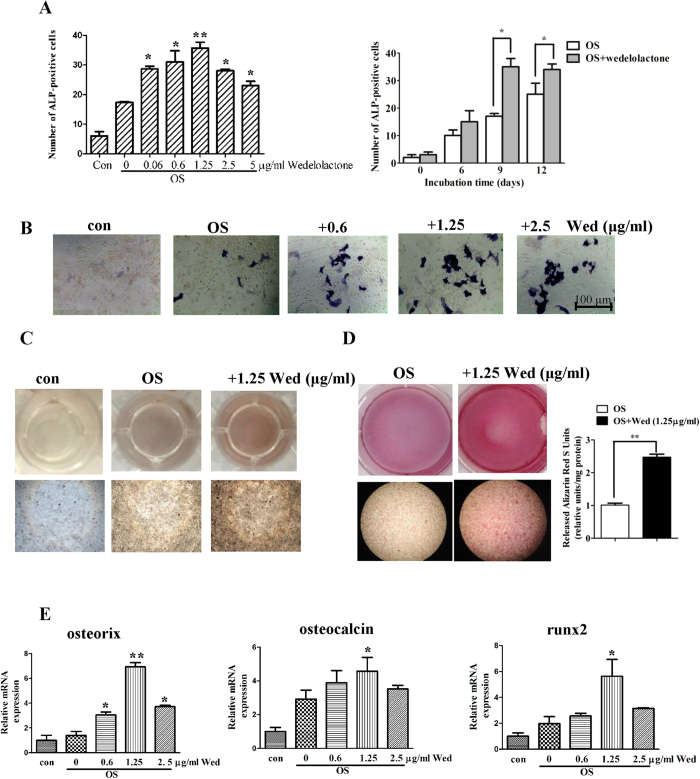
Wedeloloactone enhances osteoblastogenesis of BMSC. (**A**,**B**) Wedelolactone (Wed) increased the number of ALP staining-positive cells and ALP activity. Mouse BMSC were incubated with osteogenic medium, followed by treatment with or without the indicated doses of wedelolactone. Cells were cultured for the indicated time, and fixed for ALP staining or quantitative assay of ALP activity by using *p*-Npp as a substrate. (n = 3). Error bars denote mean ± SD. *P < 0.05, **P < 0.01 *vs.* OS treated control. (**C**,**D**) Wedelolactone enhanced bone mineralization. Mouse BMSC (5 × 10^4^ cells) were incubated with osteogenic medium followed by addition of wedelolactone for 25 d and fixed for von kossa staining or 21 d for alizarin red staining. Alizarin red S staining was quantitated by measuring the absorbance of alizarin red S released by cetylpyridinium and normalized to milligram of total protein in these cultures. (n = 3). Error bars denote mean ± SD. *P < 0.05, **P < 0.01 *vs.* OS treated control. (E) Quantitative RT-PCR analysis of osteorix, osteocalcin and runx2 expression in untreated or wedelolactone-treated BMSC. The cells were treated with or without wedelolactone for 9 d. Then total RNA was subjected to qRT-PCR (n = 3). Error bars denote mean ± SD. *P < 0.05, **P < 0.01 *vs.* OS treated control.

To examine the effects of wedeloloactone on osteoblastogenesis, we used BMSC, the osteogeninators derived from bone marrow. The BMSC obtained from BALB/c mice were cultured in an osteogenic medium (OS) containing 100 nM dexamethasone, 1 mM β-glycerophosphate, and 5 μM L-ascorbic acid 2-phosphate. The cells were incubated with various concentrations of wedeloloactone during differentiation for different days. As shown in Fig. 1A, wedeloloactone incubation for 9 d resulted in an increase in the activity of alkaline phosphatase (ALP), a marker enzyme for maturated osteoblasts, in a dose-dependent manner. 1.25 μg/ml wedeloloactone significantly increased the number of ALP-positive BMSC. 2.5 μg/ml and 5 μg/ml wedeloloactone treatment led to the decrease in the number of ALP-positive BMSC, but the number was still more than that of wedelolactone-untreated control. When the cells were incubated with 1.25 μg/ml wedelolactone for 6, 9, and 12 d, the number of ALP-positive BMSC increased with the most potent upon 9 d incubation. 1.25 μg/ml wedeloloactone increased the number of ALP-positive BMSC on the basis of observation of quantity and intensity of precipitated dye on the culture of these cells (Fig. 1B).

When wedeloloactone was administrated at the concentration of 1.25 μg/ml for 21 d, mineralization level and calcium deposits increased accordingly, as revealed by von Kossa staining (Fig. 1C) and alizarin red staining (Fig. 1D). These results suggested that wedeloloactone facilitated the differentiation of BMSC into osteoblastic cells.

### Wedeloloactone facilitates osteoblastogenesis-related marker gene expression

Marker genes, including SP7 (which encodes osterix), Bglap (encoding osteocalcin) were markedly increased in their expression during the osteogenic differentiation induced by wedelolactone especially at the concentration of 1.25 μg/ml (Fig. 1E). Runx2 has been shown to be indispensable for bone formation^22^,^23^ and a potent inducer of osteogenic differentiation^24^,^25^. 1.25 μg/ml wedelolactone treatment for 9 d strongly induced the mRNA expression of runx2. These data suggested that wedelolactone stimulated marker gene expression in osteoblastogenesis.

### Wedelolactone enhances osteoblastogenesis through the Wnt/GSK-3β/β-catenin pathway

**Figure 2:**
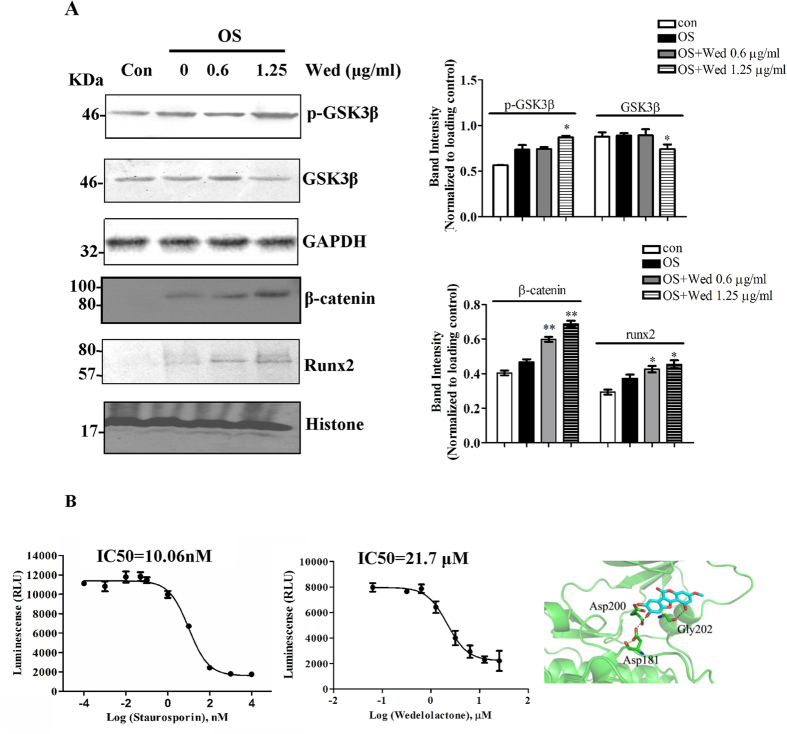
Wedelolactone regulats osteoblast differentiation through Wnt/GSK3β/β-catenin pathway. (**A**) Wedelolactone enhanced the phosphorylation of GSK3β, and nuclear accumulation of β-catenin and runx2. BMSC were incubated with or without wedelolatone for 9 d, and then cytosolic lysates and nuclear lysates were subjected to immunoblotting with antibodies as indicated. (n = 3) (**B**) The inhibitory effect of wedelolatone on GSK3β activity. Left: Different concentrations of wedelolactone or staurosporin were incubated with the reaction solution, and then GSK3β activity was assayed. Right: Cartoon representation of wedelolactone docked onto the crystal structure of GSK3β. (n = 3). Error bars (**A**,**B**) denote mean ± SD. *P < 0.05; **P < 0.01.

Wnt signaling pathways are reported to be involved in osteogenesis of BMSC. A crucial step in transducing the Wnt signal is the recruitment of a complex to the receptors, which is sequestered from a cytoplasmic complex involving glycogen synthase kinase 3 β (GSK3β), subsequently inhibits β-catenin phosphorylation, thereby stabilizing β-catenin^26^. Therefore, inhibition of GSK3β facilitates activation of Wnt signaling pathway. Figure 2A showed that GSK3β phosphorylation was up-regulated and GSK3β expression was slightly downregulated in the presence of 1.25 μg/ml wedelolactone, indicating that GSK3β activity was inhibited by wedelolactone. The control of the nuclear β-catenin level is critical for the Wnt/β-catenin signaling pathway. The nuclear accumulation of β-catenin was up-regulated in wedelolactone-treated BMSC. The nuclear accumulation of runx2 was also increased. Further, we examined the directly inhibitory activity of GSK3β by wedelolactone. GSK3β activity was inhibited by wedelolactone at 0.1, 1.25, 2.5, 5 μg/ml (Fig. 2B), suggesting that GSK3β is the target of wedelolactone for enhancing osteogenesis. However, compared with staurosporin, a GSK3β inhibitor, of which IC50 is 10.06 nM, the IC50 value for wedelolactone was higher, and was determined to be 21.7 μM. This result suggested that the inhibitory effect of wedelolactone on GSK3β activity was weak. A possible binding cavity for wedelolactone on GSK3β was determined by using the AutoDock Vina Docking program and the structure of GSK3β (Protein Data Bank, PDB) as the binding molecule. The structure of GSK3β-wedelolactone revealed that a group of residues in the cavity could make contacts with wedelolactone through electrostatic or hydrophobic interactions (Fig. 2B).

### Wedelolactone inhibits osteoclastic activity and function

**Figure 3:**
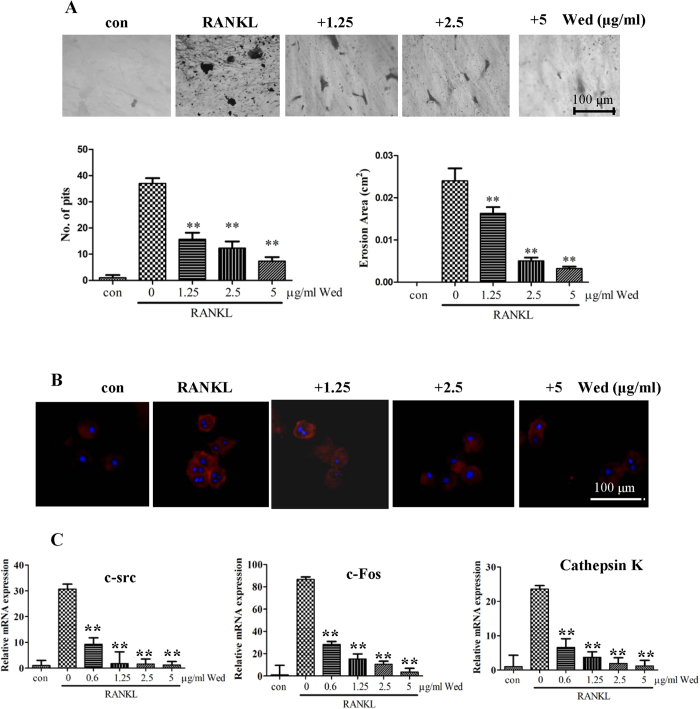
Wedelolactone inhibits bone resorption in RAW264.7 cells. (**A**) Wedelolactone suppressed formation of bone resorption pits. RAW264.7 cells (1 × 10^5^ cells) were treated with 100 ng/ml RANKL for 4 d and then incubated with or without wedelolactone on dentine slices for 2 d. Dentine slices were stained with Mayer’s hematoxylin after removal of cells. The resorption pits were visualized with light microscopy. The numbers of pits and erosion areas were analyzed with Image-Pro Plus software (bottom). (n = 3). Error bars denote mean ± SD. *P < 0.05, **P < 0.01 *vs.* RANKL-treated control. (**B**) Wedelolactone suppressed RANKL-induced actin ring formation in RAW264.7 cells. RAW264.7 cells (1 × 10^5^ cells) were incubated with RANKL for 4 d and then treated with wedelolactone for 2 d. Cells were fixed and stained for F-actin. (**C**) Wedelolactone inhibited mRNA levels of c-src, c-fos and cathepsin k. Wedelolactone was pretreated with or without wedelolactone for 6 d in the presence of RANKL. Cell lysates or total RNA was subjected to qRT-PCR. (n = 3). Error bars denote mean ± SD. **P < 0.01 *vs.* RANKL-treated control.

Our previous study showed that wedelolactone inhibited osteclastic RAW264.7 proliferation and differentiation^14^. In this study, we further investigated the effect of wedelolactone on osteoclast function. When RAW264.7 cells were cultured on dentin slices, RANKL-induced mature osteoclast caused the resorption of lacunae and the formation of pits, as compared to blank slice controls. In contrast, the number and area of pits on the surface of the dentin slices induced by RANKL were markedly decreased after incubation with different concentrations of wedelolactone for 2 d (Fig. 3A). In the presence of RANKL, RAW264.7 cells were differentiated into mature osteoclasts and formed obvious actin-ring structures, a character of mature osteoclasts during osteoclastogenesis^27^, by FITC-phalloidin staining. However, the size and the number of actin-ring structures were significantly reduced when the cells were treated with 1.25 to 5 μg/ml wedelolactone (Fig. 3B), suggesting that wedelolactone suppressed the formation of the actin-ring in osteoclastic RAW264.7 cells.

### Wedelolactone inhibits osteoclastogenesis through NF-κB/c-fos/NFATc1 pathway

The binding of RANKL to its receptor RANK results in activation of tumour-necrosis factor (TNF) receptor-associated factor 6 (TRAF6), which stimulates the NF-κB pathway^28^,^29^,^30^. RANKL also activates the activator protein1 (AP-1) transcription factor complex, including c-Fos, which cooperates with NF-κB to induce NFATc1, thus activating the transcription of osteoclast-specific genes such as Ctsk, Acp5 and Nfatc1^31^,^32^,^33^.

**Figure 4:**
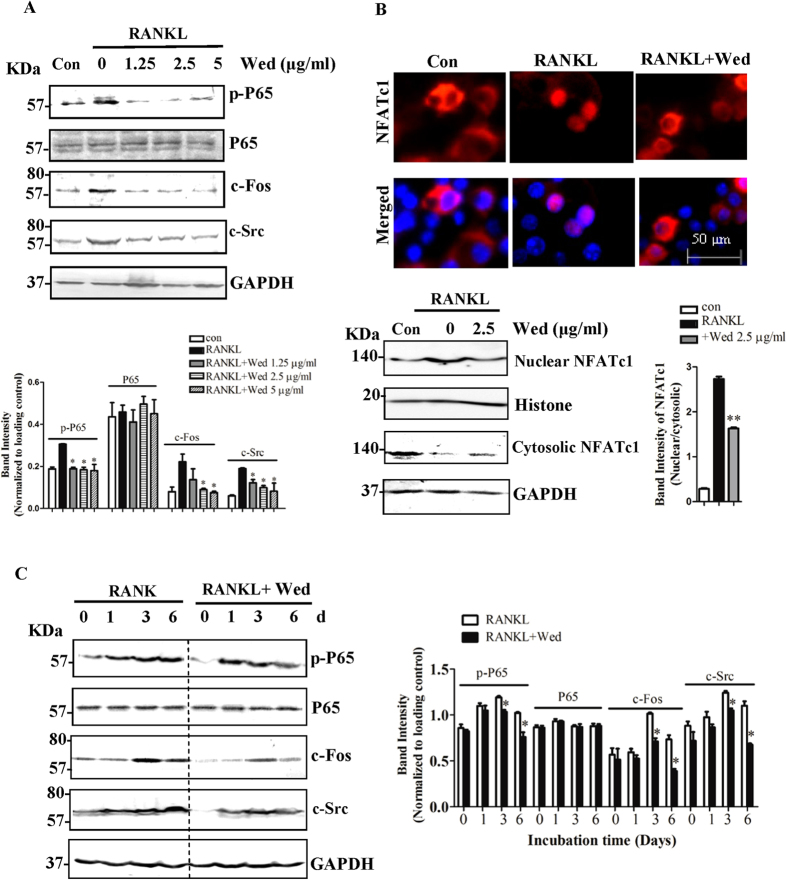
Wedelolactone inhibits RANKL-induced NF-κB/NFATc1 pathway. (**A**) Wedelolactone inhibited phosphorylation of p65, and suppressed c-Fos as well as c-src expression stimulated by RANKL. RAW264.7 cells were incubated with or without wedelolactone and then stimulated with 30 ng/ml RANKL for 6 d. The cell lysates were extracted and subjected to Western blot analysis with the indicated antibodies. (n = 3). (**B**) Wedelolactone inhibited RANKL-induced NFATc1 nuclear translocation. RAW264.7 cells were incubated with or without wedelolactone and then stimulated with 30 ng/ml RANKL for 6 d. The location of NFATc1 was visualized by immunofluorescence analysis (Magnification ×400) and quantified by Western blot analysis.(n = 3) (**C**) Wedelolactone inhibited phosphorylation of p65, and suppressed c-Fos as well as c-src expression after 1, 3, 6 d incubation with RANKL. RAW264.7 cells were incubated with or without wedelolactone and then stimulated with 30 ng/ml RANKL for indicated time. The cell lysates were extracted and subjected to Western blot analysis with the indicated antibodies. (n = 3). Error bars (A–C) denote mean ± SD. *P < 0.05; **P < 0.01.

The expression levels of osteoclast differentiation related marker genes of c-Src, c-fos and cathepsin K, were reduced in the presence of different concentrations of wedelolactone by qRT-PCR assays (Fig. 3C). Using Western blot assays, we verified that wedelolactone inhibited RANKL-induced phosphorylation of NF-κB/p65 (Fig. 4A). The expression of c-fos and c-Src in total proteins was increased in RANKL-treated RAW264.7 cells, whereas wedelolactone treatment significantly downregulated the level of c-fos and c-Src. NF-κB induces the initial induction of NFATc1, the expression of which is autoamplified by NFATc1 in cooperation with c-fos^18^,^31^. To determine whether wedelolactone regulates the activation of NFATc1, we performed immunofluorescence staining to determine the nuclear translocation of NFATc1. In the absence of RANKL, most NFATc1 was located in the cytoplasm. Upon 6 d RANKL stimulation, NFATc1 was translocated into the nucleus. However, the nuclear translocation of NFATc1 was blocked when incubation with 2.5 μg/ml wedelolactone and RANKL together. Western blot assays, as shown in Fig. 4B, revealed that the upregulated expression of NFATc1 in the nucleus was downregulated in the presence of wedelolactone. Upon stimulation with RANKL at different time point, the upregulated phosphorylation of NF-κB/p65 and expression of c-fos and c-Src were reversed by wedelolactone (Fig. 4C).

### Wedelolactone exerts osteoprotective effect by inhibiting osteoclast activity and stimulating osteoblast differentiation *in vivo*

**Figure 5:**
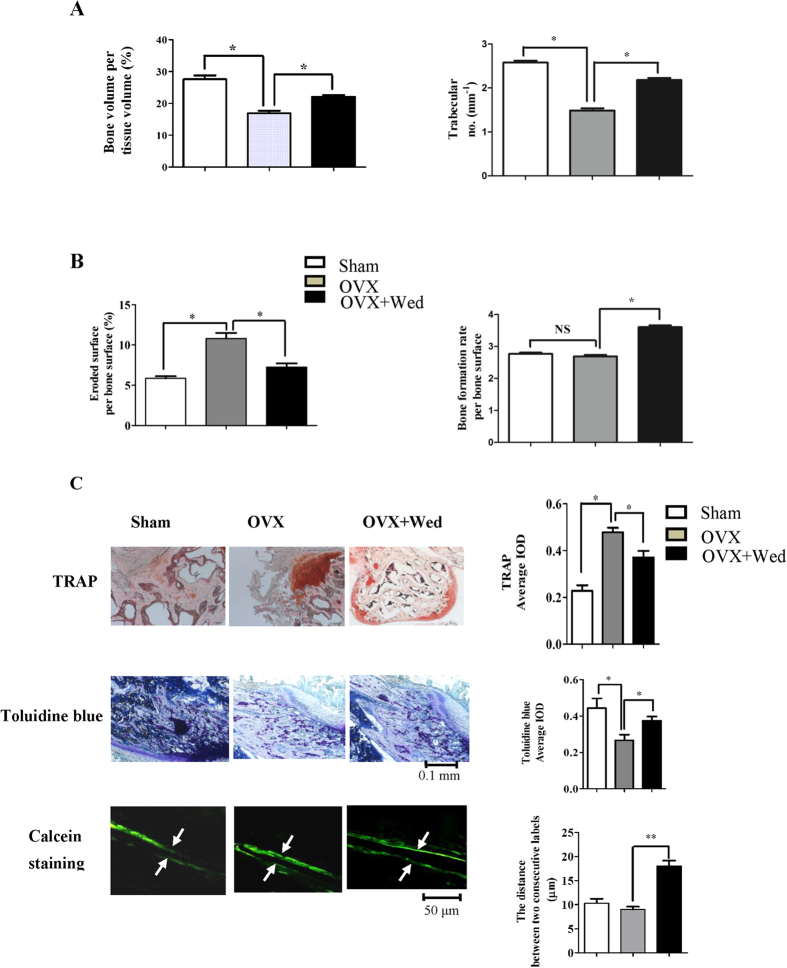
Wedelolactone prevents ovariectomy-induced bone loss by inhibiting osteoclast activity and enhancing osteoblast activity. (**A**) Bone histomorphometric analysis of trabecular bones from 2 month-old female mice. Bone volume/tissue volume and trabecular number was analyzed with osteoMeasure Analysis System. (n = 5–6). (**B**) Eroded surface/bone surface for osteoclastic bone resoption and bone formation rate per bone surface were analyzed. (n = 5–6). NS: no significance. (**C**) Histological analysis of the femur of sham-operated (Sham), ovariectomized (OVX) and wedelolactone-treated OVX mice (n = 4–6). Up: TRAP staining of the proximal tibiae of Sham, OVX and wedelolactone-treated OVX mice. Middle: Toluidine blue staining of the proximal tibiae of Sham, OVX and wedelolactone-treated OVX mice. Bottom: New bone formation was determined by calcein double labeling. Arrows mark distance between calcein-labelled layers. TRAP staining and Toluidine blue staining were quantified by average IOD, and the distance between two consecutive labels in the proximal tibiae was quantified with Image-Pro Analyzer 6.2 software (Media Cybernetics, Bethesda, MD, USA). Error bars (**A**–**C**) denote mean ± SD. *P < 0.05; **P < 0.01.

To determine the *in vivo* effect of wedelolactone on bone metabolism, an ovariectomized mouse model was created as described^34^. Ovariectomized 9-week-old mice were intraperitoneally injected with wedeloloactone every two days starting two days after ovariectomy and continuing for four weeks. Figure 5A showed a markedly decrease in bone volumn and trabecular number at the femur after ovarietomy. Treatment of OVX mice with wedelolactone significantly prevented the VOX-induced bone loss, as measured by different parameters. To investigate whether wedelolactone prevented OVX-induced bone loss by inhibiting osteoclast activity and simultaneously stimulating osteoblast activity, bone morphometric analysis of the osteoclastic parameters in the femur was performed. Figure 5B showed that eroded surface/bone surface was increased dramatically in OVX mice, whereas this parameter decreased in wedelolactone-treated OVX mice. The observation of TRAP staining on long bone of each group showed that wedelolactone administration reduced TRAP activity induced in OVX mice (Fig. 5C).

The effect of wedelolactone on bone formation *in vivo* was further investigated. Histomorphometric analysis revealed that bone formation rate per bone surface in OVX mice had no change compared with sham controls. Treatment of OVX mice with wedelolactone significantly increased this osteoblastic parameter (Fig. 5B). The osteoblast activity by toluidine blue staining was enhanced in the presence of wedelolactone. Also, the distance between two consecutive labels by calcein double staining increased after treatment with wedelolactone (Fig. 5C), indicating that the new bone formation was enhanced by wedeloloactone administration.

## Discussion

Bone remodeling consists of bone formation and bone resorption phases. These two phases are under the control of coupling factors such as RANKL, which link the bone resorption with formation, thus maintaining bone homeostasis. Due to the couple of these two processes, one target for drugs, such as bisphosphonates, which inhibit farnesyl pyrophosphate synthase, thereafter decrease bone degradation, but also reduce bone formation. Likewise, drugs such as parathyroid hormone increase bone synthesis in patients, but bone degradation also rise. Therefore, it is attractive to explore the new class of dual action therapetutic agents for osteoporosis, which can inhibit bone resorption and promote bone formation, synchronously. Traditional Chinese medicine is a successful source of therapeutic agents and drug leads. *Ecliptae herba* has been used for thousands of years in China to nourish bones. *Ecliptae herba* extracts are shown to be effective in treating bone metabolism of OVX rats^8^. However, the bioactive constituents and mechanisms remain largely unknown. Our previous study showed that wedelolactone, a compound from *Ecliptae herba*, inhibited osteoclastic RAW264.7 proliferation and differentiation. The effect was similar to previous report that wedelolactone inhibited breast cancer-induced osteoclastogenesis^35^. In this study, we for the first time demonstrated that wedelolactone had the ability to regulate bone remodeling by synchronously inhibiting bone resorption and promoting bone formation. Wedelolactone played a critical role in the bone formation phase, in which osteoblasts were differentiated from BMSC, and subsequently extensively produced bone mineralization. At the same time, wedelolactone restrained the formation of ostoclastic bone resorption pits from resorbing the formed bone. Of note, we observed that the enhanced ALP activity and osteoblast marker gene expression was the most potent when 1.25 μg/ml wedelolactone was used, while the stimulatory activity decreased with exposure to the increasing doses of wedelolatone, as shown in Fig. 1. This observation indicated that the effective dose range of wedelolacone for enhancement of osteogenesis was narrow. Thus, optimization of the chemical structure of wedelolactone or development of its analogues might contribute to the extended stimulatory activity for osteogenesis.

Using an ovariectomized mouse model^34^, we further confirmed through histomorphometric analysis that wedelolactone prevented bone loss by suppressing eroded surface/bone surface and TRAP activity *in vivo*. Also, the enhanced effects of wedelolactone on bone formation were confirmed by investigating osteoblast differentiation and osteoblast activity using OVX mice. New bone formation by calcein labeling was observed to be increased by treatment with wedelolactone. The action of wedelolactone on bone homeostasis might be distinct with the widely used osteoporosis drugs, such as bisphosphonates and PTH_1–34_, which inhibit or stimulate both processes at the same time^6^,^36^. This dual functional effect of wedelolactone is fascinating and its mechanism might be distinct with current drugs.

The canonical Wnt/GSK3β/β-catenin signaling is a key pathway for regulating bone formation and contributing to osteoblastic differentiation^22^,^37^. A crucial step in tranducting the Wnt signal is to destroy the cytoplasmic GSK3β complex by inducing GSK3β phosphorylation, and subsequently, inhibits β-catenin phosphorylation, thereby stabilizing β-catenin. The accumulated β-catenin thus enters the nucleus and activates the expression of the Wnt target genes. In this study, phosphoration of GSK3β was elevated by wedelolactone, and following the nuclear translocation of β-catenin was enhanced in the presence of wedelolactone, as shown in Fig. 2. The expression of GSK3β was slightly downregulated by wedelolactone. It is suggested that the Wnt/β-catenin signaling was activated in the wedelolactone-enhanced osteogenesis. Runx2 is the master osteogenic transcription factor that takes part in the process of osteoblast maturation. Runx2 is also found to transduce Wnt-signaling for mediating osteogenic differentiation of BMSC^38^. It can act as crosstalking regulator between Wnt signaling pathways and others that enhance osteogenesis. Wedelolactone treatment facilitated nuclear expression of runx2, and osteoblast marker gene expression, indicating that wedelolactone induced Wnt/GSK3β/β-catenin pathway-mediated runx2 activation, resulting in enhanced osteoblastogenesis.

Binding of RANKL to its receptor RANK results in the recruitment of the adaptor molecules tumor necrosis factor receptor-associated factors (TRAFs) and c-src. The NF-κB signaling pathway is one of the key downstream signaling pathways from the complex^19^,^20^. Wedelolactone had significant inhibitory effect on the phosphorylation of NF-κB subunit p65. RANKL also activates the activator protein 1 (AP-1) transcription factor complex, including c-Fos, which cooperates with NF-κB to induce NFATc1, thus activating the transcription of osteoclast-specific genes. Previous study reported that wedelolactone inhibited Akt/mTOR signaling in breast cancer-induced osteoclastogenesis^35^. Akt signaling acts the upstream of NF-κB. Combined with the current results suggested that wedelolactone might influence Akt/mTOR/NF-κB signaling pathway, subsequently inhibiting NFATc1 activation, resulting in suppression of osteoclastogenesis. In this study, wedelolactone decreased the expression of c-Fos as well as the translocation of NFATc1, suggesting that wedelolactone modulated the NF-κB/c-Fos/NFATc1 signaling pathway in RANKL-induced osteoclastogenesis. NFATc1 can regulate the expression of a number of genes associated with osteoclast differentiation and function. As a result, wedelolactone inhibited the expression of osteoclastogenesis related marker genes, including c-Fos, cathepsin K, and c-Src. Cathepsin K, as a cysteine protease, is selectively expressed by osteoclasts and can degrade bone matrix proteins. Elimination of cathepsin K in RAW264.7 cells by wedelolactone resulted in inhibition of bone resorption. c-Src is related to osteoclast migration and therefore inhibition of c-Src by wedelolactone might block RAW264.7 cell migration and thereby reducing osteoclast activity. All of these results suggested that wedelolactone had inhibitory effect on the RANKL-induced osteoclastogenesis through NF-κB signaling pathway. In addition, wedelolactone at 2.5 μg/ml was verified to markedly inhibit NFATc1 nuclear localization and RANKL/RANK signaling pathway. Besides, 1.25 μg/ml wedelolactone, of which dose osteoblast formation was enhanced, possibly have a role in suppressing those osteoclast related signaling pathways, since osteoclastic differentiation and function was inhibited by 1.25 μg/ml wedelolactone.

**Figure 6:**
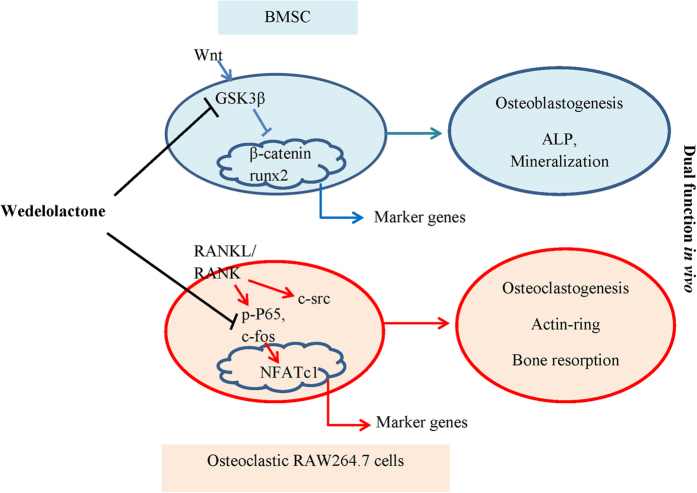
Graphical representation of the dual functional role of wedelolactone in osteoblastogenesis and osteoclastogenesis.

Previous studies reported that the treatment of lithium, an inhibitor for both GSK3β enzymatic activity and inositol metabolism circuits, increased bone mass in animals with age-related osteoporosis and oophorectomy-induced osteoporosis^39^,^40^,^41^. In this study, we found that wedelolactone directly inhibited the activity of GSK3β, suggesting that wedelolactone was an inhibitor of GSK3β (Fig. 6). We further confirmed that wedelolactone was docked onto the crystal structure of GSK3β through electrolstatic or hydrophobic interactions, which was distinct with staurosporin^42^. In addition, wedelolactone is reported to inhibit IκBα phosphoration^43^, consistent with the result of this study. These data indicated that wedeloloactone was a potent multi-target active compound (Fig. 6). Further study of competitive binding of wedelolactone with two targets or multi-targets corresponding for regulating bone resorption and bone formation would be interesting. Besides, according to the chemical structure of wedelolactone, wedelolactone is the derivation of coumarin, which might have the estrogenic activity and interact with estrogen receptors. Further study of targets for wedelolactone will be needed.

Collectively, our data for the first time demonstrated that wedelolactone enhanced osteoblastogenesis and simultaneously suppressed osteoclastogenesis both *in vitro* and *in vivo*. Furthermore, the resluts showed that wedelolactone facilitated osteoblastogenesis through activation of Wnt/GSK3β/β-catenin signaling pathway, which led to the activation of runx2 and the expression of downstream genes. Simultaneously, wedelolactone inhibited osteoclastogenesis through inhibition of RANKL/RANK/NF-κB pathway, resulting in suppression of c-Fos/NFATc1 activation and osteoclast marker gene expression.

## Materials and Methods

### Materials

Wedelolactone was provided by Key Laboratory of Separation Science for Analytical Chemistry at Dalian Institute of Chemical Physics, Chinese Academy of Sciences (Dalian, China). The purity was above 98%. p65, phospho-p65, c-Src, and NFATC1 antibodies were purchased from Santa Cruz Biotechnology (Santa Cruz, CA, USA); DAPI was bought from Abcam (Cambridge, MA, USA). TRIZOL reagent was purchased from Invitrogen (Carlsbad, CA, USA); Phalloidin–Atto 565, RANKL were bought from Sigma (St Louis, MO, USA); ALP staining kit was obtained from Sigma.

### Osteoblastogenesis assay

For *in vitro* osteoblast differentiation, mouse bone marrow stromal cells were isolated from 8-week old BALB/c mice according to a previously published protocol, and were cultured with α-MEM with 10% FBS. After 5 days, cells were reseeded (1 × 10^4^ per cm^2^) and cultured with osteogenic medium (100 nM dexamethasone, 1 mM β-glycerophosphate, and 5 μM L-ascorbic acid 2-phosphate). Culture medium was changed every third day. After nine days, ALP staining and activity measurement were performed, and after 21 days, bone nodule formation was assessed by alizarin red staining as described previously^44^. For quantification, the alizarin red S–stained cultures were further incubated with 100 mM cetylpyridinium chloride for 1 h to solubilize and release calcium-bound alizarin red into solution^45^. Mineral deposition was determined with von Kossa staining on day 25.

### *In vitro* osteoclastogenesis assay

Mouse pre-osteoclastic RAW264.7 cells were purchased from the Type Culture Collection of Chinese Academy of Sciences (Shanghai, China).The cells were plated at 1 × 10^4^ per cm^2^ in DMEM supplemented with recombinant RANKL. For drug assays, wedelolactone was added at different concentrations to the culture medium. Then cells were incubated at 37 °C with 5% CO_2_ in a humidified incubator, and fed daily with RANKL-supplemented medium for the indicated days. The cell pellet was collected for Western blot analysis or quantitative real-time RT-PCR analysis.

### Actin ring formation and pit assay

After stimulation with 100 ng/ml RANKL for 4 d, the cells were treated with different concentrations of wedelolactone for 2 d. Then we fixed the cells with 4% paraformaldehyde for 10 min and washed them three times with PBS. Cells were dehydrated with acetone and permeabilized with 0.1% Triton X-100 in PBS for 10 min. Then the cells were incubated with 50 mg/ml Phalloidin–Atto 565 for 40 min, the actin ring assay was performed as described previously^46^.

For the resorption pit assay, RAW264.7 cells were seeded on dentine slices and treated with 100 ng/ml RANKL for 4 d prior to incubation with wedelolactone for 2 d. After the culturing period, osteoclasts were removed from the dentine slices by ablation, using cotton tips, and the dentine slices were stained with Hematoxylin Solution (Sigma). Images were acquired with a light microscope at a 100*magnification, and areas of resorption pits were analyzed with Image-Pro Analyzer 6.2 software (Media Cybernetics, Bethesda, MD, USA)^47^,^48^.

### Immunofluorescence staining

The effect of wedelolactone on nuclear translocation of NFATc1 was examined by immunofluorescence, as described previously^49^. Briefly, Cells were fixed in 4% paraformaldehyde and then permeabilized with 0.25% Triton X-100 in PBS. Cells were blocked in PBS containing 10% goat serum. Primary antibodies used were NFATc1 (1:100); Fluorescent signals were detected using an Olympus Ix51 inverted fluorescent microscope with 40x objective.

### GSK3β activity assay

GSK3β kinase assay was performed by the ADP-Glo^TM^ kinase assay kit (Promega, Madison, USA). Briefly, different concentrations of wedelolactone were incubated with GSK3β, 1 μg GSK3β substrate, and 50 μM ATP at room temperature for 60 min. Then 25 μl ADP-Glo^TM^ reagent was added and incubated at room temperature for 40 min. 50 μl kinase detection reagent was added and incubated for another 60 min. Luminescence (integration time 1 second) was recorded with Multi-Mode Detection Platform (Molecular Devices, Austria).

### Western blot analysis

For western blot analysis, cells were resuspended in buffer A (10 mM HEPES, pH 7.9, 1.5 mM MgCl_2_, 10 mM KCl, 0.5 mM DTT, 10 mM NaF, 2 mM Na_3_VO_4_, 1 mM pyrophosphoric acid and Complete TM protease inhibitors) and incubated on ice for 10 min. Cells were then centrifuged at 700 g at 4 °C for 10 min. The supernatant was collected as the cytosolic fraction. For nuclear protein extraction, the pellet from the 700 g centrifugation was washed by buffer A, resuspended in buffer B (20 mM HEPES, pH 7.9, 1.5 mM MgCl_2_, 420 mM NaCl, 0.2 mM EDTA, 10 mM NaF, 2 mM Na_3_VO_4_, 1 mM pyrophosphoric acid and Complete TM protease inhibitors) and incubated on ice for 5 min. The protein concentration in the cell lysates was determined using Bradford Protein Assay. Western blot analysis was performed as previously described^48^ using the antibodies above.

### Quantitative real-time RT-PCR

All work was carried out in a designated PCR-clean area. RNA was extracted from cells using Trizol reagent (Gibco-BRL, Rockville, Md.) and isolated as specified by the manufacturer. The RNA was DNAse-treated (DNase I-RNase-Free, Ambion) to remove any contaminating DNA; 200 ng of total RNA was reverse-transcribed with oligo dT primers using the High Capacity cDNA RT Kit (Applied Biosystems) in a 20-μl cDNA reaction, as specified by the manufacturer. For quantitative PCR, the template cDNA was added to a 20 μl reaction with SYBR GREEN PCR Master Mix (Applied Biosystems) and 0.2 μM of primer. The amplification was carried out using an ABI Prism 7000 for 40 cycles under the following conditions: an initial denaturation of 95 °C for 10 min, plus 40 cycles of 95 °C for 15 s, then 60 °C for 1 min. The -fold changes were calculated relative to β-actin using the ΔΔCt method for c-Fos, c-src, Cathepsin K and mRNA analysis. The following primer sets were used: mouse Cathepsin K: forward, 5′-CTTCCAATACGTGCAGCAGA-3′; reverse, 5′-TCTTCAGGGCTTTCTCGTTC-3′; mouse NFATc1: forward, 5′-TGGAGAAGCAGAGCACAGAC-3′; reverse, 5′-GCGGAAAGGTGGTATCTCAA-3′; mouse β-actin: forward, 5′-GTACGCCAACACAGTGCTG-3′; reverse, 5′-CGTCATACTCCTGCTTGCTG-3′. mRunx2, forward, 5′-GCCGGGAATGATGAGAACTA-3′; reverse, 5′- GGTGAAACTCTTGCCTCGTC-3′; mOsteocalcin: forward, 5′-GCCATCACCCTGTCTCCTAA-3′; reverse, 5′-GCTGTGGAGAAGACACACGA-3′; mOsterix: forward, 5′-GGAGGTTTCACTCCATTCCA-3′; reverse, 5′-TAGAAGGAGCAAGGGGACAGA-3′.

### Ovariectomized mouse model and bone histomorphometric analysis

We created an ovariectomized mouse model as described^34^. All procedures on mice were carried out following guidelines and protocols reviewed and approved by the Ethics Committee at Dalian Medical University. Briefly, nine-week-old C57BL/6 female mice were ovariectomized or sham operated. More than five mice were examined in each group. 2 days after ovariectomy mice were divided into three groups of six mice each: sham operated mice (Sham), ovariectomized mice treated with vehicle (OVX) and OVX mice treated with wedelolactone. 10 mg/kg wedelolactone was injected intraperitoneally into the ovariectomized mice every two days. Sections of femurs and lumbar vertebrae were obtained for histomorphometric analysis using the OsteoMeasure Analysis System (Osteometrics, Decatur, GA, USA) according to standard criteria. Three-dimensional microcomputed tomography analyses and bone morphometric analyses were performed as described^50^,^51^.

For osteoclast TRAP staining, toluidine blue staining 34, and calcein staing 34, proximal tibiae was isolated and fixed in 10% paraformaldehyde fixation buffer (PFA) and decalcification performed with 10% EDTA for 2 weeks. The samples were embedded in paraffin for staining (Sigma). The quantitative analysis was carried out using the “measure integrated optical density” (IOD) function of Image Pro-Plus version 6.0 and average IOD was used for statistical analysis.

### Statistical analysis

All experimental data are presented as the mean ± SD, with values from more than three experiments. Student’s t-test was used to compare the difference between two means. *P < 0.05, **P < 0.01.

## Additional Information

**How to cite this article**: Liu, Y.-Q. *et al*. Wedelolactone enhances osteoblastogenesis by regulating Wnt/β-catenin signaling pathway but suppresses osteoclastogenesis by NF-κB/c-fos/NFATc1 pathway. *Sci. Rep.* **6**, 32260; doi: 10.1038/srep32260 (2016).
